# Interfacial Transcrystallization and Mechanical Performance of 3D-Printed Fully Recyclable Continuous Fiber Self-Reinforced Composites

**DOI:** 10.3390/polym13183176

**Published:** 2021-09-18

**Authors:** Manyu Zhang, Xiaoyong Tian, Dichen Li

**Affiliations:** State Key Laboratory for Manufacturing Systems Engineering, Xi’an Jiaotong University, Xi’an 710049, China; 18153200156@163.com (M.Z.); dcli@mail.xjtu.edu.cn (D.L.)

**Keywords:** continuous fiber self-reinforced composites, 3D printing, transcrystallization, mechanical properties, fully recyclable

## Abstract

To fully exploit the preponderance of three-dimensional (3D)-printed, continuous, fiber-reinforced, thermoplastic composites (CFRTPCs) and self-reinforced composites (which exhibit excellent interfacial affinity and are fully recyclable), an approach in which continuous fiber self-reinforced composites (CFSRCs) can be fabricated by 3D printing is proposed. The influence of 3D-printing temperature on the mechanical performance of 3D-printed CFSRCs based on homogeneous, continuous, ultra-high-molecular-weight polyethylene (UHMWPE) fibers and high-density polyethylene (HDPE) filament, utilized as a reinforcing phase and matrix, respectively, was studied. Experimental results showed a qualitative relationship between the printing temperature and the mechanical properties. The ultimate tensile strength, as well as Young’s modulus, were 300.2 MPa and 8.2 GPa, respectively. Furthermore, transcrystallization that occurred in the process of 3D printing resulted in an interface between fibers and the matrix. Finally, the recyclability of 3D-printed CFSRCs has also been demonstrated in this research for potential applications of green composites.

## 1. Introduction

Fiber-reinforced polymer composites (FRPCs) are often used in the aerospace and automotive industries and in manufacturing sporting goods and biomedical implants requiring stiff lightweight materials [1,2,3]. FRPCs are usually composed of two constituents, the reinforcement and the matrix materials, which inevitably leads to interfacial and material recycling problems. This is considered to be a major drawback of FRPCs, because composite mechanical properties are greatly influenced by the interfacial interaction between reinforcement fibers and the matrix [4,5]. On the other hand, the recycling process requires isolating the fiber and matrix, which results in various drawbacks such as degraded materials and negative environmental impact besides being expensive. Constant efforts have been made in preparing better recyclable FRPCs to enhance their efficient reprocessing [6,7]. Unlike traditional fiber-reinforced composites made up of different constituents, self-reinforced composite (SRC) materials consist of matrix phases and reinforcing, which are composed of the homogeneous material that belongs to the same family of polymers but shows different structures and performances. The identical chemical properties as well as crystal morphologies of the components in this composite system make the compatibility better on the interface. Moreover, SRCs are fully recoverable by reprocessing, thereby providing environmentally friendly materials in accordance with environmental legislation [8,9,10].

The SRC technique, which was first raised in 1975 by Capiati and Porter [11], entails embedding gel-spun high-modulus polyethylene (PE) fibers in lower-melting-point PE resin. SRCs not only exhibit excellent interfacial affinity, but also do not require additional processing steps to separate the reinforcement elements and matrix components during the recycling process. On account of these benefits, it has been subsequently applied to many other polymers. Various thermoplastic polymers, PE, polypropylene, polyethyleneterephthalate, polymethyl methacrylate, polyamide, and bio-based polymers have so far been used to design SRCs [12,13,14,15]. The most widely used method for the preparation of fiber SRCs is the hot compaction of fibers. This method results in fibers’ partial surface melting, whereas the melted part of fibers forms as the matrix of the self-reinforced composites after cooling. Because the fiber interior does not melt, the reinforcement remains highly oriented. The major drawback of this technique is that, because the resin matrix and the reinforcement are made of the same material, the processing window as regards temperature does not exceed several degrees and even the slightest overheating of the fibers inevitably degrades its reinforcing properties. Production by film stacking was implemented to widen the processing window of fiber SRC preparation. However, all these conventional fiber SRC processes involved inextricable drawbacks including costly molds and time-consuming processes, making it difficult to fabricate complex composite structures and hampering the extensive use of SRCs [16].

The 3D printing of continuous fiber-reinforced thermoplastic composites (CFRTPCs) introduced by Tian et al. [17] inspired the proposed concept of producing continuous fiber self-reinforced composites (CFSRCs) by 3D printing in this study. Although 3D-printed carbon-fiber-reinforced composites have been widely studied, they exhibit poor interfacial adhesion, resulting in poor mechanical performance owing to the limited impregnation pressure and time in the 3D-printing process [18,19,20]. Some researchers have systematically reported printing prepreg filaments about continuous fiber-reinforced composites from MarkForged Inc. (Rostock, Germany) [21,22,23]. In contrast, the SRC interface usually consists of crystalline superstructures heterogeneously formed at the surface of the fibers. The morphological characteristics (just as the alleged transcrystallization of the matrix material onto fiber surfaces) perhaps are related to improving stress transfer ability along the fiber–matrix interface [24]. Therefore, the combination of SRCs and 3D-printed continuous fiber-reinforced composites can not only improve the interface but also enable rapid manufacturing of lightweight, recyclable, and environmentally friendly composites.

In this study, the 3D printing of continuous ultra-high-molecular-weight polyethylene (UHMWPE) fiber self-reinforced high-density polyethylene (HDPE) composites was studied. The essential printing temperature of CFSRCs was investigated by differential scanning calorimetry (DSC). The impact of printing temperatures on the mechanical properties of the UHMWPE fibers was tested to establish a qualitative correlation between mechanical performance and temperature. Furthermore, the effect of 3D printing temperature on the mechanical properties of CFSRCs was studied to obtain the appropriate performance parameters. The specimens’ microstructures were observed using scanning electron microscopy (SEM), and the transcrystalline behavior of homogeneous fibers and the matrix during the 3D-printing process was investigated by polarized optical microscopy (POM). Finally, the recyclability of the printed composites was determined by analyzing the DSC and melting flow index (MFI) results. A closed-loop recycling mode of self-reinforcing composites can be realized by 3D printing.

## 2. Materials and Methods

### 2.1. Materials and Equipment

HDPE filaments with a diameter of 1.75 mm were used as matrix materials. They were supplied by Tailong Corp. (Taizhou, China). UHMWPE fibers (580 fibers in a bundle, with tenacity of about 337.27 N/tex and a density of about 0.97 g/cm^3^) from Sovetl Corp. (Dongguan, China) were used as reinforcement. A 3D printer (COMBOT-200) from Fibertech Corp. (Xi’an, China) was utilized as the experimental equipment.

### 2.2. 3D-Printing Process

**Figure 1:**
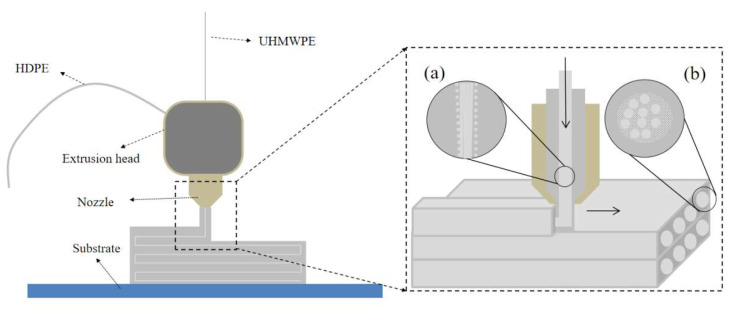
3D printing scheme for CFSRCs with UHMWPE fibers on an HDPE matrix. (**a**) Partial surface melting of UHMWPE fibers. (**b**) Recrystallization of UHMWPE fibers.

A sketch of the 3D-printing process for CFSRCs is displayed in Figure 1. Continuous UHMWPE fibers entered the nozzle from the fiber coil throughout the inner hole of the extrusion head. Meanwhile, the resin matrix filament was delivered into the extrusion head and then heated to a liquid state in the nozzle. Consequently, the continuous fiber was impregnated in the molten matrix inside the nozzle and then extruded out as SRCs from the nozzle orifice. As the resin-coated extruded fiber was printed on the substrate surface and subsequently on top of the previously printed layer, it quickly solidified and adhered to the substrate and the previously solidified layer, respectively; therefore, the fibers were continuously drawn out by the solidified fiber inside the SRC. An important additional feature of SRCs is that partial surface melting of initial fibers occurs in the inner hole of the extrusion head (Figure 1a) and recrystallization as a matrix occurs after cooling (Figure 1b) during the 3D-printing process. In order to reduce the warpage of CFSRCs during the printing process, the print bed heated to 90 °C. Because of the crystallization of the HDPE, the CFSRCs exhibited negligible warpage.

### 2.3. Experiments

Thermal analysis of UHMWPE fibers and the HDPE matrix was performed using a differential scanning calorimeter (Mettler Toledo DSC1) in an argon atmosphere according to ASTM D3417-83. Briefly, specimens were heated up to 200 °C at a heating rate of 10 °C/min and cooled to 25 °C; the UHMWPE fibers were reheated at the same rate of 10 °C/min up to 200 °C.

Individual UHMWPE fiber bundles impregnated with the HDPE matrix were extruded horizontally onto the print bed at a constant height with a constant gauge length of 50 mm to establish a correlation between mechanical performance and printing temperature. Aluminum alloy reinforcing sheets were arranged in two sections of individual filaments to reduce tensile stress concentration. Then the individual filaments specimen was made to assure the whole specimen was kept at the baseline. Finally, epoxy resin glue was used to bond the aluminum alloy-reinforced sheet (Figure 3a).

Tensile tests of individual filaments were conducted at a speed of 5 mm/min using a universal testing machine (CMT4304-5kN). The CFSRC specimens with a size of 180 mm × 15 mm × 2 mm were tensile-tested on the same universal testing machine at a speed of 5 mm/min according to GB/T1447-2005 (ISO:527-4:1997) [25]. Each experimental group printed at different temperatures was tested. Five samples were tested for each experimental group to obtain an average value. For all samples, the mechanical properties were tested parallel to the orientation of fibers. Unnotched impact tests of the simply supported beam of 3D-printed CFSRCs compared with HDPE were carried out. The CFSRC specimens (80 mm× 10 mm × 4 mm) were tested on the pendulum impact testing machine (China, XJJ-50) according to GB/T1043-1993 (ISO 179-1982) [26]. In addition, all the CFSRC specimens were printed with a 1.0 mm nozzle diameter and the print bed heated to 90 °C. All the CFSRC specimens were printed at a hatch spacing of 1.0 mm, a layer thickness of 0.3 mm, and a printing speed of 120 mm/min for various temperatures.

Surface morphologies were conducted using a Hitachi SU-3500 electron microscope. Samples were prepared by cutting using liquid nitrogen or etched using xylene for 2 h at 80 °C. Experience proves that different reagents are required to etch samples and present different detail. The samples were prepared by cutting using liquid nitrogen to get the cross-section SEM images. The samples were etched with xylene for engraving. The samples were etched by a mixture reagent for investigation of the transcrystallization. The mixture reagent contained 1 volume of distilled water, 4 volumes of orthophosphoric acid, 10 volumes of sulfuric acid, and 1% wt/vol potassium permanganate. The interfacial morphology of the CFSRC specimen was observed using an Olympus BX51 POM.

## 3. Results

### 3.1. Process Temperature for 3D Printing

**Figure 2:**
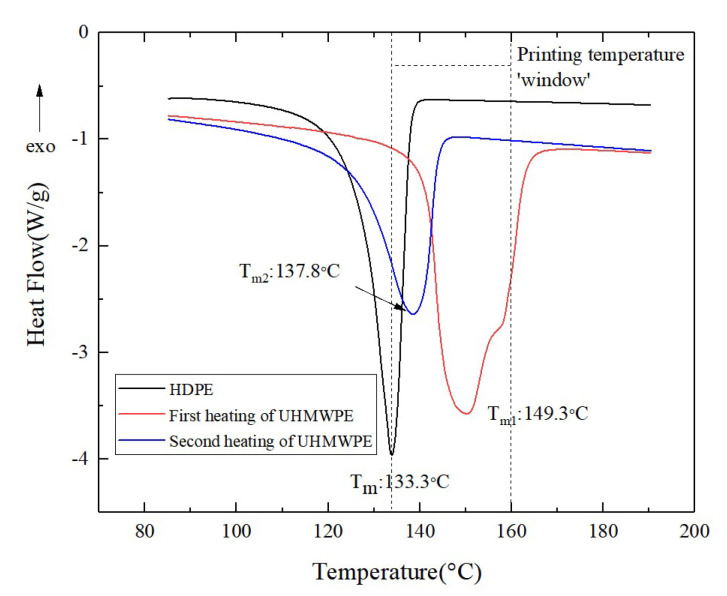
DSC thermograms of UHMWPE and HDPE.

Temperature is a crucial process parameter for the printing of CFSRCs and has a remarkable impact on the melting state of the fiber and matrix. A specific feature of SRCs is the chemical similarity of the fiber and the matrix [22]. The different physical states of composite constituents would cause significant differences in their melting points, resulting in a processing temperature “window”. The HDPE matrix and UHMWPE fiber DSC curves showed that HDPE and UHMWPE melted at 133.3 °C and 149.3 °C, respectively, as shown in Figure 2. The printing temperature window was extended above the fiber melting point, on account of UHMWPE fibers coated with HDPE resin forming sheath-core in the 3D-printing process. This may allow surface melting of the fiber without degrading the mechanical performance of the highly oriented core, as seen from Figure 1a. The melted portion of the fiber formed a matrix of SRCs with an isotropic structure; it then recrystallized after cooling, as shown in Figure 1b. Moreover, the volume of melted UHMWPE fibers can be changed by varying the temperature during the 3D-printing process. An increasing printing temperature results in a decrease in oriented core fibers by progressive melting until they completely melt. In the 3D-printing process, the fibers were pulled out of the nozzle while subjected to a constraint. The constraining of the fibers makes it possible to retain highly oriented fibers at a high printing temperature [12]. Further experiments of progressive heating during the 3D-printing process gave the upper limit to the printing temperature window of the UHMWPE–HDPE composite as ~160 °C.

### 3.2. Mechanical Properties of 3D-Printed CFSRCs

**Figure 3:**
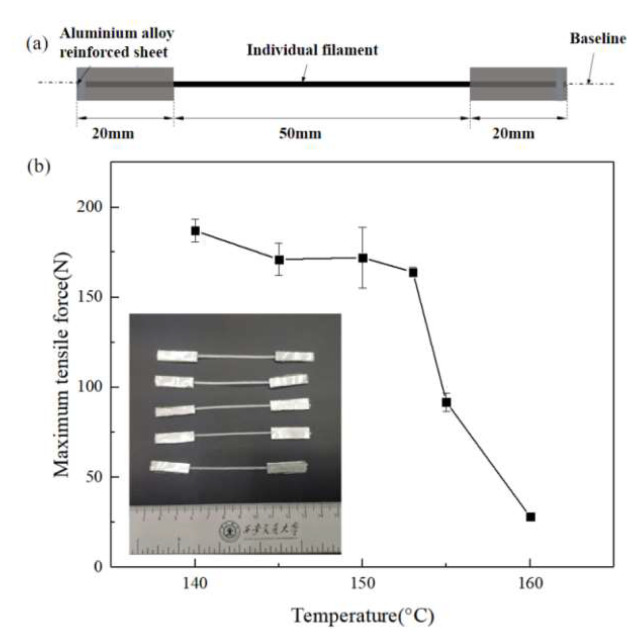
(**a**) Individual filament tensile test specimen. (**b**) Mechanical properties of single UHMWPE fiber bundles obtained at various temperatures.

The mechanical properties of 3D-printed CFRCs depend on the reinforcement fibers and the interface. The highly oriented structure of UHMWPE fibers acts as the principal force-bearing component in CFSRCs. The number of oriented chains in the fibers will directly affect its mechanical properties. However, the oriented chains may relax at high temperatures; therefore, UHMWPE fibers may exhibit molecularly disoriented polymer chains [12]. The second heating DSC curve generated for the UHMWPE fibers revealed a melting peak corresponding to 137.8 °C (Figure 2), compared to a melting peak corresponding to 149.3 °C in the first heating curve. The oriented structure relaxation happened as the first heating process exceeded the fibers’ melting point, causing degradation of the fibers’ mechanical properties. Consequently, the maximum force that the individual filaments can withstand was used as a criterion for retaining or losing the oriented fibers’ structure. The influence of the printing temperature on the oriented UHMWPE fibers’ structure was studied using the maximum tensile force. From the curve shown in Figure 3, it can be concluded that the mechanical properties change insignificantly at printing temperatures lower than the melting point (149.3 °C) but deteriorate dramatically at temperatures above 150 °C. This can be qualitatively described by the increase in the melted portion of the UHMWPE fibers as the printing temperature increases. Hence, loss of the oriented phase occurred after the melting point until 160 °C, where the UHMWPE became isotropic.

**Figure 4:**
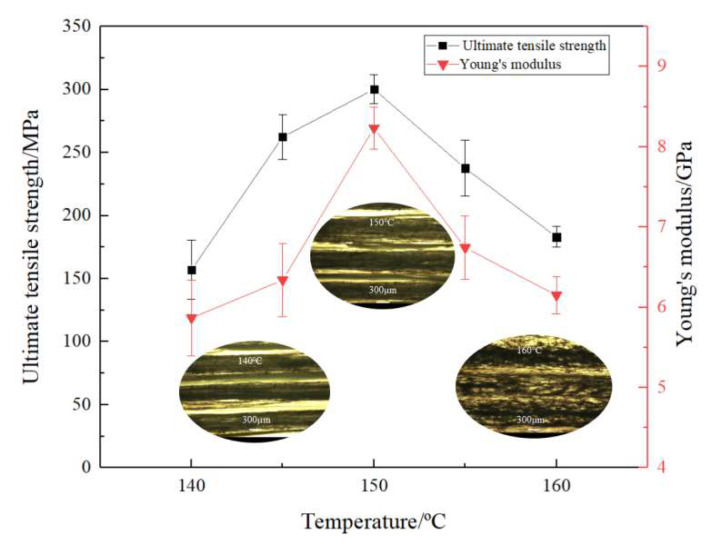
Mechanical properties of the CFSRCs printed at various temperatures.

Tensile tests were conducted to evaluate the impact of the printing temperature on mechanical properties such as tensile strength as well as Young’s modulus of CFSRCs (Figure 4). Tested specimens were printed at a hatch spacing of 1.0 mm, a layer thickness of 0.3 mm, and a printing speed of 120 mm/min for various temperatures (140 °C, 145 °C, 150 °C, 155 °C, and 160 °C). The tensile strength of CFSRCS printed at 150 °C achieved 300.2 MPa, with Young’s modulus of >8 GPa, whereas for the printed HDPE, the tensile strength and Young’s modulus were as low as 19 MPa and 0.73 GPa, respectively. Therefore, the tensile strength of 3D-printed CFSRCs is 15 times higher than that of the HDPE matrix. In addition, the data showed that both Young’s modulus and tensile strength of CFSRCs increased as the printing temperature increased until the fiber melting temperature (149.3 °C). This correlation can be qualitatively explained by the fact that the MFI of HDPE increased, improving the flowability of the matrix into the UHMWPE fiber bundle as well as increasing interlayer adhesion and thus enhancing the mechanical properties.

**Figure 5:**
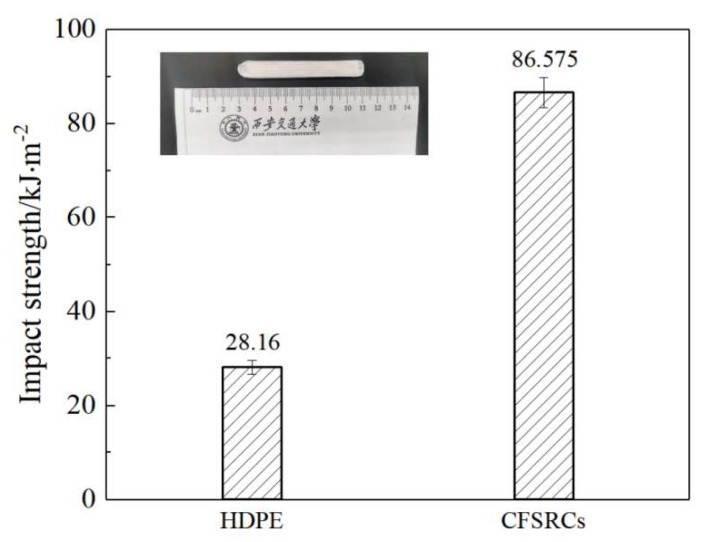
The impact strength of 3D-printed CFSRCs and HDPE.

The tensile strength and Young’s modulus of CFSRCs both decreased as the printing temperature exceeded the fiber melting point (149.3 °C). This behavior is related to the relaxation of the oriented structure of UHMWPE fibers, resulting in the decrease of reinforcing elements and, consequently, decrease of mechanical properties. It was confirmed by the optical microscopy of the monolayer of CFSRCs that the oriented UHMWPE fiber bundles become amorphous at temperatures higher than the melting point of UHMWPE fibers. The higher the printing temperature, the more the proportion of oriented UHMWPE fibers was reduced, which coincided with the mechanical properties. The impact strength is shown in Figure 5, where the 3D-printed CFSRCs at 150 °C were 86.575 kJ/m^2^ and the 3D-printed HDPE was 28.16 kJ/m^2^. The impact strength of CFSRCs was about 3 times higher than HDPE, which indicated good toughness.

**Figure 6:**
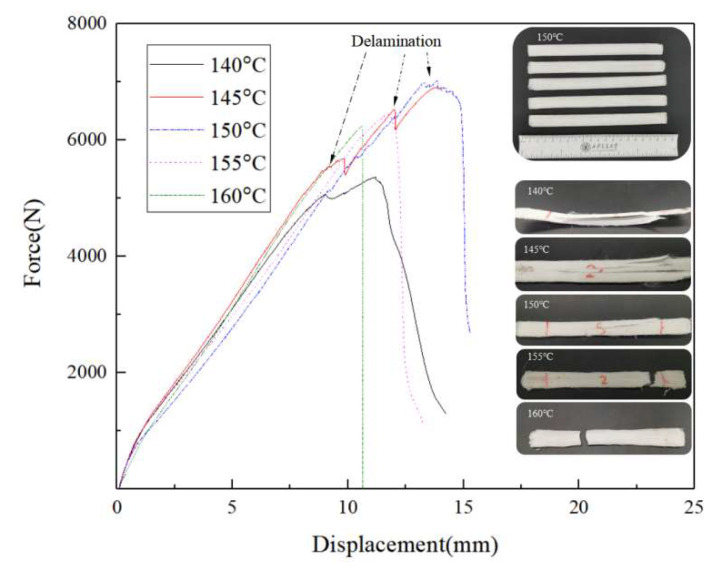
Force and displacement curves of CFSRCs obtained at various temperatures.

Furthermore, zigzag patterns emerged in the force–displacement curves of CFSRCs (Figure 6) when the printing temperature was below the UHMWPE fiber melting point (149.3 °C), indicating that the composite had hierarchically delaminated during loading. By increasing the printing temperature from 140 °C to 150 °C, stronger interlayer adhesion helped to prevent delamination through crack-arresting mechanisms and enhanced the stress transfer between filaments, thereby doubling the tensile strength. Simultaneously, the fracture mode of samples printed at different temperatures changed from delamination to brittle failure, which is consistent with the displacement–force curves.

### 3.3. Microstructures of 3D-Printed CFSRCs

**Figure 7:**
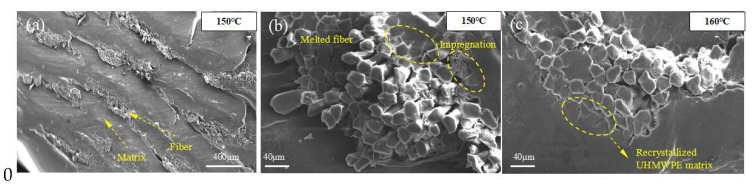
SEM images of the CFSRC’s surface cut perpendicular to the fibers’ axis printed at (**a**,**b**) 150 °C and (**c**) 160 °C.

SEM images of the sample’s surface, which is vertical to the fiber axis (Figure 7a), showed the unique ability of 3D printing to additively develop architectures layer by layer. It can also be observed that UHMWPE fibers were packaged in the HDPE matrix and extruded from the nozzle to form a monolithic material. Our previous research demonstrated that the interfaces of 3D-printed CFRCs involved two aspects: Interfacial impregnation and interfacial bonding. Interfacial impregnation results from the melted resin matrix flowing into the fiber bundle, which ensured that single fibers are connected to each other by the resin matrix [24]. Effective impregnation can not only prevent inter-void formation but also increases the apparent contact area between fibers and the resin matrix.

**Figure 8:**
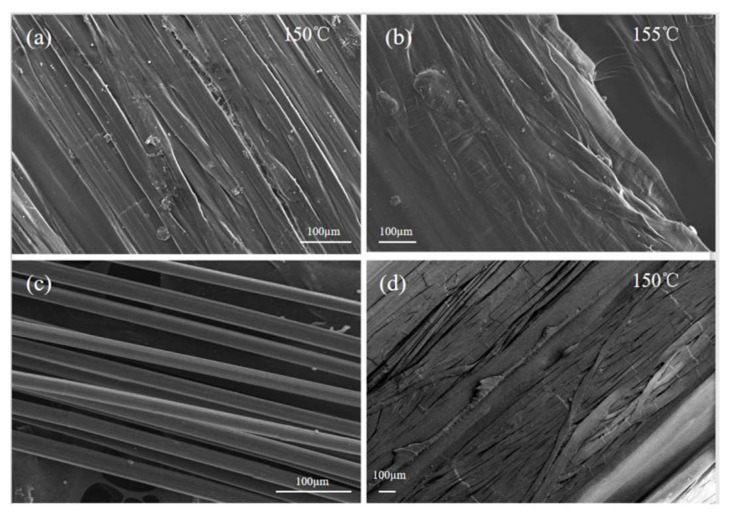
SEM images of the CFSRC’s surface: Longitudinal section of tested samples printed at (**a**) 150 °C and (**b**) 155 °C; (**c**) initial UHMWPE fibers and (**d**) those etched with dimethylbenzene.

It can be observed from Figure 7b that the surface of the UHMWPE fiber bundle impregnated with HDPE resin or melted fibers recrystallized as the isotropic UHMWPE matrix connected the fibers with each other. This is one of the advantageous features of SRCs compared with conventional composites. The increase in fiber –matrix and fiber–fiber contact areas resulted in better stress transfer to the reinforcing elements, consequently improving mechanical properties. Under the same scale length, the oriented UHMWPE fibers’ proportion noticeably decreased. As a result, the mechanical properties of the fibers were degraded markedly, which is consistent with previous mechanical tests. Owing to the high temperature and pressure of the extrusion nozzle, the transverse section of the fibers became nearly elliptical (Figure 7c). By increasing the printing temperature from 150 °C to 155 °C (Figure 8a,b), the UHMWPE fibers in CFSRCs began to curl, indicating that it was progressively harder for the melted resin matrix to flow into the interior of the fiber bundle due to the increasing of temperature. At the same time, the fibers’ oriented supramolecular structure was destroyed by the higher temperature, as can be observed by fibers curling up or the intense melting of the fibers at the macroscopic level. As the dimethylbenzene dissolved the surficial HDPE matrix from the CFSRCs, flaky fibers emerged. Furthermore, the initial UHMWPE fibers (Figure 8c) subjected to hot pressure during the printing process become flaky fibers. Moreover, grooves had clearly formed between the fibers, indicating that the HDPE matrix had sufficiently impregnated the UHMWPE fiber bundle (Figure 8d).

## 4. Discussion

### 4.1. Transcrystallization in the 3D-Printed CFSRCs

As previously mentioned, the other aspect of the interfaces in 3D-printed CFRCs that is worth mentioning is the bonding between fibers and the matrix. The identical chemical property as well as the crystalline morphology of the components’ CFSRC system result in their mutual compatibility at the interface. This generates not only a relatively fine interfacial adhesion, but also a distinct fiber–matrix interface, in which the resin matrix crystallizes on the fiber surface to produce transcrystallization. Recent studies have shown that the transcrystallization of the resin matrix grown from fiber surfaces may enhance the stress transfer capability between the fiber–matrix. This is a process in which the resin matrix crystallizes from the surface of the fiber and develops only in a direction perpendicular to the outer surface of the fiber, thus producing a transgranular cylindrical interfacial layer [27,28]. Although the microscopic details of transcrystallization, as well as its processing condition dependence, are well documented, the literature contains limited information about the influence of 3D-printing conditions on transcrystalline interphases.

**Figure 9:**
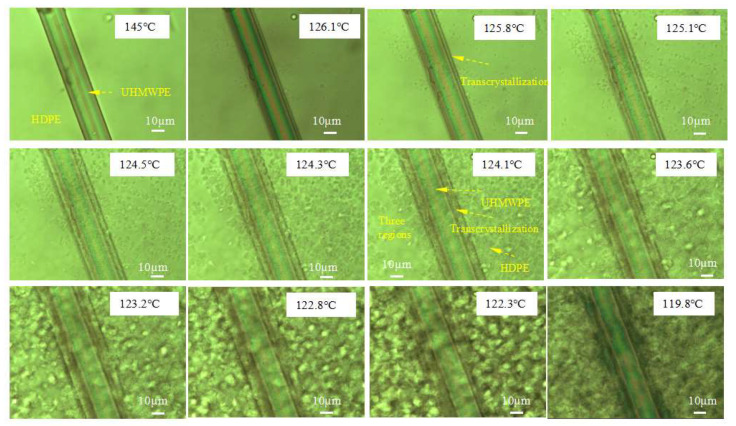
POM experiment on the single UHMWPE fiber–HDPE composite at various temperatures.

**Figure 10:**
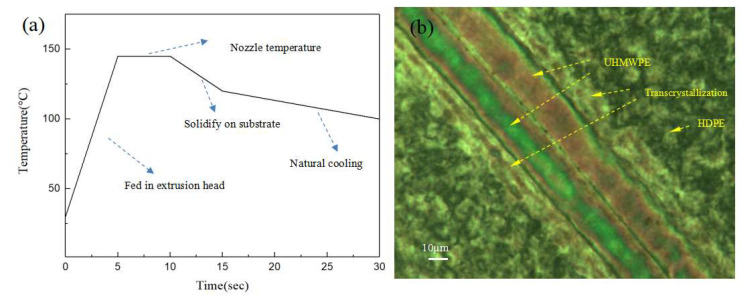
(**a**) Simulation of the 3D-printing temperature field at a printing speed of 120 mm/min. (**b**) POM images of two UHMWPE fiber–HDPE composite specimens.

**Figure 11:**
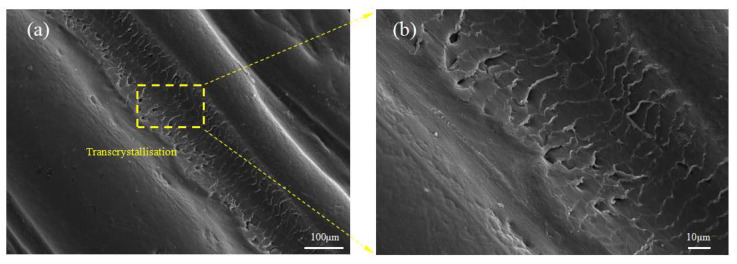
SEM image of a longitudinal section etched by potassium treatment of CFSRCs. (**a**) SEM image of transcrystallization in CFSRCs. (**b**) Details of transcrystallization in CFSRCs.

The formation of transcrystalline UHMWPE fibers on the HDPE matrix was investigated by POM under a changing temperature field during the 3D-printing process. As shown in Figure 9, transcrystallization can be observed in a direction vertical to the fiber surface on account of the nucleation that occurred at an adequately high density alongside the fiber surface when the temperature decreased (Video S1). The sample of the single UHMWPE fiber-reinforced HDPE exhibited three different regions: The UHMWPE fiber, the HDPE, and transcrystallization. The UHMWPE fiber was embedded in a transcrystallization layer surrounded by the HDPE resin matrix that consisted of spheruliticsuper structures. The image presented proves that the growth of transcrystalline superstructures is possible during the 3D-printing process. The changing temperature field during the 3D-printing process was simulated as shown in Figure 10a. There was a temperature rise period when the fibers and matrix were fed into the extrusion head, then the temperature remained constant for about 10s, and they solidified out of the nozzle. Simultaneously, transcrystallization can be observed as growth from two UHMWPE fibers, as shown in Figure 10b. A transcrystalline layer of 3D-printed SRCs appeared after etching with the permanganic reagent in Figure 11. The transcrystalline layer was composed of sheet-like superstructures. Furthermore, their growth direction was normal to the fiber axis, as described earlier. Obvious lamellae crystalline structures were observed between the fiber and the matrix, which is consistent with previous simulation results.

**Figure 12:**
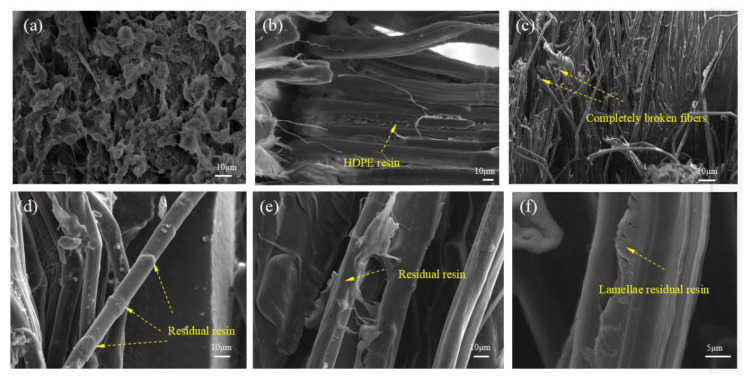
Microstructures of fractured surfaces of SRCs after tensile tests at a printing temperature of 150 °C. (**a**) Fractured surface of the HDPE matrix. (**b**,**c**) Longitudinal section of the tested samples. (**d**–**f**) Detail of fractured UHMWPE fibers.

Figure 12a characterizes the spherulitic supramolecular structure of the HDPE matrix fracture surfaces, thereby demonstrating the isotropic properties of the HDPE matrix. Figure 12b indicates that the UHMWPE fibers were well impregnated into the matrix. Completely broken fibers and split fibers are also visible in Figure 12c. Moreover, there is a residual resin matrix on the surface of the pull-out fibers observed in Figure 12d, thereby demonstrating the good interfacial adhesion between UHMWPE fibers and the HDPE matrix. This should enhance the stress transfer between the fibers and the matrix, thereby improving the tensile behavior of loaded CFSRCs. A residual resin layer on the fibers and lamellae residual resin are predominantly observed after the samples fractured, as shown in Figure 12e,f, which are expected to arrest further propagation of the cracks that acted as stress concentrators.

### 4.2. Recoverability of 3D-Printed CFSRC Material

**Figure 13:**
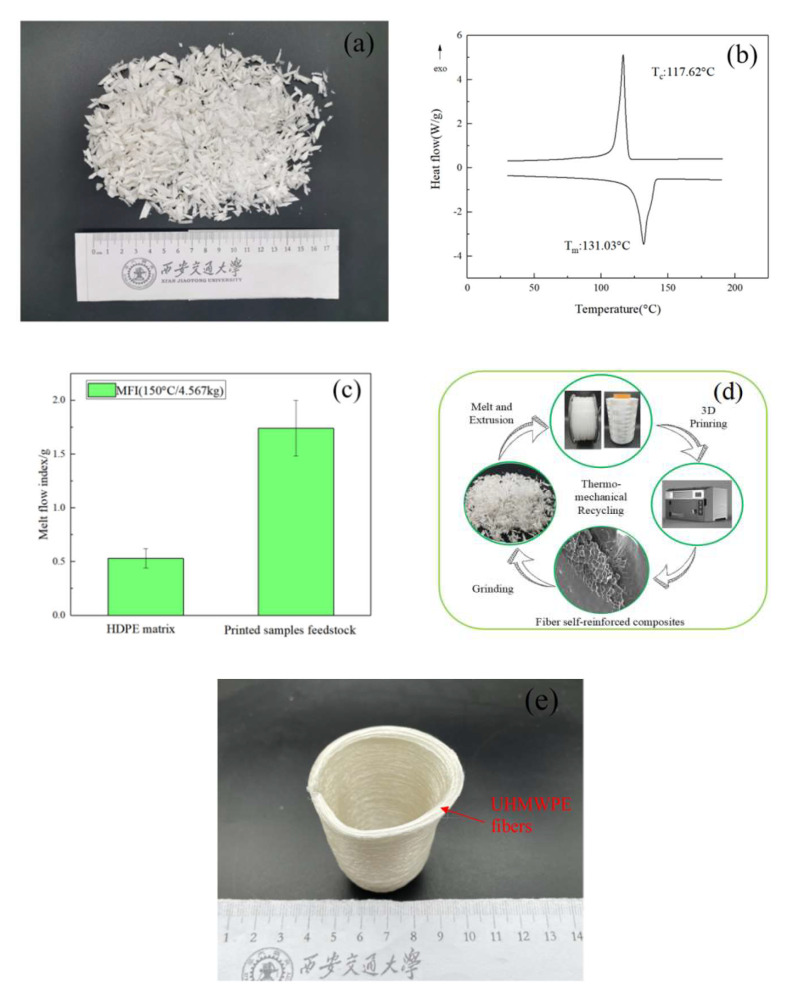
(**a**) Recycled printed samples as feedstock material. (**b**) DSC curves of recycled material. (**c**) MFI of HDPE and recycled material. (**d**) Closed-loop recycling. (**e**) Medical below-knee prosthetics model printed with UHMWPE fibers and HDPE matrix.

To demonstrate the recoverability of 3D-printed CFSRC materials, the printed samples were crushed for the application to feedstock material, as shown in Figure 13a. As shown in Figure 13b, the DSC analysis of the recycled CFSRCs revealed one distinct melting peak at 131.03 °C, indicating that the oriented UHMWPE fibers had already melted into isotropic non-oriented UHMWPE, and had blended with the HDPE matrix. The melting peak of UHMWPE/HDPE blends was insignificantly lower than the pure HDPE, therefore we inferred that the addition of UHMWPE affects the crystal integrality of HDPE. The MFI quantifies the fluidity of the material at the indicated temperature and load. The MFI of the recycled CFSRCs at a temperature of 150 °C was ~1.74 g/min, which was markedly higher than that of pure HDPE at the same temperature owing to the added UHMWPE shown in Figure 13c. The UHMWPE fibers subjected to a high temperature caused molecular disorientation of the UHMWPE chains, and the entanglement degree of HDPE molecular chains was reduced with the blend of UHMWPE chains. As a consequence, this material is ready to be fully recycled on account of its high melt flow index. Based on these results, the process of closed-loop recycling shown in Figure 13d can be proposed. However, the molecular weight of the recycled printed CFSRCs, and their corresponding performance, cannot be ignored in future research.

### 4.3. Potential Application Areas

As for the potential application areas, the following application scenarios can be suggested in the future. UHMWPE fiber has high strength and a high modulus, as well as wear resistance. Based on the mechanical performance study, the tensile strength, as well as the impact strength of CFSRCs, proved to be substantially raised. The achieved results set the next investigation plan. Self-reinforced composites fabricated through 3D printing of continuous UHMWPE fibers promise the achievement of high mechanical performance, impact resistance in the military industry, and biomedical devices. For instance, CFSRCs could be applied in ballistics as an armor vest due to the excellent energy absorption performance. Compared with the traditional hot compaction of CFSRCs, 3D printing could optimize the fiber arrangement and fiber trajectory of CFSRCs to achieve the best bulletproof effect. CFSRCs are particularly important in biomaterials applications. For example, it can be used as a knee replacement implant since any additives composed of different chemicals could affect biocompatibility. What is more, the free-form 3D-printing method can be customized for different people to satisfy a clinical effect. Figure 13e shows a real-time application of CFSRCs as a medical below-knee prosthetic thanks to the impact resistance. Moreover, 3D printing can achieve complex structures and personalized customization.

## 5. Conclusions

3D printing of CFSRCs was studied in this study. The correlation between 3D-printing temperature and the mechanical properties of CFSRCs based on a UHMWPE fiber-reinforced HDPE matrix was investigated. The results showed that the tensile properties present peak values at approximately the melting temperature of UHMWPE fibers, achieving an optimum tensile strength of 300.2 MPa as well as Young’s modulus of 8.2 GPa. A printing temperature lower than the UHMWPE fibers’ melting point results in weak bonding between layers and poor impregnation among the matrix and fiber bundles. By contrast, at a printing temperature exceeding the melting point of UHMWPE fibers, relaxation can take place. Meanwhile, molecular disorientation of the polymer chains may be observed. Relaxation of the oriented phase will bring about the degradation of the fibers’ mechanical properties, and consequent degradation of the mechanical properties of CFSRCs. Transcrystallization occurs between UHMWPE fibers and the HDPE matrix in the process of 3D printing. This has a considerable effect on the transfer of stress from fibers to the matrix when subjected to loading. Finally, the printed samples confirmed their potential to be fully recycled, and the performance of the recycled feedstock material should be investigated in future research. The potential application areas of CFSRCs include bulletproof vests and medical below-knee prosthetics.

## Data Availability

All data is available in the main text or in the Supplementary Materials. Further details can be obtained from the corresponding author upon reasonable request.

## Supplementary Materials

The following are available online at https://www.mdpi.com/article/10.3390/polym13183176/s1, Video S1: Transcrystallization evolution.
